# Factors Influencing the Perceived Economic Benefits of Innovative Agri-Environmental Contracts

**DOI:** 10.1007/s00267-024-02027-8

**Published:** 2024-08-11

**Authors:** Tracy Bradfield, Kina S. Harmanny, Thia Hennessy, Catharina J. E. Schulp

**Affiliations:** 1 Cork University Business School, University College Cork, Cork, Ireland; 2 Vrije Universiteit Amsterdam, Institute for Environmental Studies, Environmental Geography Group, De Boelelaan 1111, 1081HV Amsterdam, the Netherlands

**Keywords:** Agri-environmental climate public goods, AECPG, Results-based contracts, Contract design, Environmental policy, Sustainable agriculture, code: Q54, Q57, Q58

## Abstract

Continued innovation in contract design may enhance the delivery of agri-environmental climate public goods (AECPG), but barriers to adoption arise in terms of how farmers perceive the economic benefits. Therefore, this paper examines survey data from Ireland and the Netherlands to analyse whether land managers agree that results-based, collective action, value chain and land tenure contracts for the delivery of AECPG are understandable, applicable to their farm and economically beneficial. Using Probit models, we also identify groups of land managers who perceive the different contract types as being economically beneficial, and these findings can inform policymakers of farmer groups who need adequate consideration during the design of agri-environmental contracts. For example, greater incentives could encourage older farmers to enrol in results-based contracts in Ireland and value chain contracts in the Netherlands. We also find a link between contract duration and the perceived economic benefits of collective action contracts in both countries, with land managers in Ireland desiring a longer duration. We highlight that policymakers and land managers in Ireland could apply lessons from the design of agri-environmental contracts in the Netherlands, where they are more common and varied. Greater knowledge exchange between users and non-users of such contracts would also help bridge the gap between theory and practice in both countries.

## Introduction

The European Union (EU) is increasingly utilising agri-environmental policy to encourage land managers to protect and support ecosystems which are crucial for both society and long-term sustained agricultural production (Cullen et al. 2020). The Common Agricultural Policy (CAP) has three clear environmental goals, each of which are reflected in the European Green Deal and Farm to Fork strategy. These include addressing climate change, protecting natural resources, and enhancing biodiversity. A goal of the Green Deal is to achieve net zero greenhouse gas emissions across the EU by 2050 (European Commission 2023). In addition to the strengthening of Agri-Environmental Schemes (AES), the EU has commissioned work that aims to design innovative and more efficient contracts within these schemes.^1^

To date, agri-environmental contracts^2^ have primarily been input- or action-based (McGurk et al., 2020). These contracts reward land managers for taking on a task rather than encouraging them to focus on its results. However, the European Commission is expressing greater interest in AES that award payments based on environmental outcomes (Burton and Schwarz 2013; Chaplin et al. 2021). The need for enhanced incentives for sustainable land management has led to the development of innovative contract types where payments are based on individual farms’ environmental results (*results-based contracts*), outcomes of collective action on a landscape scale (*collective action contracts*), the market/customer paying a higher price to reimburse or reward farmers for adopting environmental practices (*value chain contracts*), and land lease agreements that include environmental clauses (*land tenure contracts*). Continued innovation in contract design may make AES more effective in the delivery of agri-environmental climatic public goods (AECPG), but barriers to adoption arise in terms of how farmers perceive the benefits. To date, literature has focused on the determinants of adoption, including Gatto et al. (2019), Lastra-Bravo et al. (2015), McGurk et al. (2020), Paulus et al. (2022) et cetera, but not on the specificity of perceived economic benefits. It is important that this is studied because, as noted by White and Hanley (2016), if farmers do not feel properly compensated for their provision of environmental goods and services, the payments fail to induce a socially desirable level of adoption.

Therefore, the objective of this research is two-fold. First, to understand if land managers (farmers or forestry managers) agree that results-based, collective action, value chain and tenure contracts for the delivery of AECPG are understandable, applicable to their farm and economically beneficial. These innovative contracts incentivise farmers to increase the provision of AECPG alongside private goods (Prager 2015) and they are experimental in that they have not been a core feature of traditional AES (Bredemeier et al. 2022). In theory, these new contract types can provide benefits for land managers and the environment but there is little empirical evidence on their acceptance by land managers. Therefore, by assessing the understandability, applicability, and perceived economic benefits of these contracts, we investigate the potential barriers to their acceptance. In summary, to design effective agri-environmental contracts, it is important to understand how they will be received by the key decision-makers, the land managers.

Second, we assess the characteristics of land managers who agree that a contract type is, or would be, economically beneficial for them, as economic benefits are identified in the literature as a strong determinant of increased adoption (Bradfield et al. 2024; Cullen et al. 2021, Pavlis et al. 2016; Vermundt et al. 2022; Wilson and Hart 2000, et cetera). We do this by applying a Probit model to survey data. The views of different types of land managers on AES design need to be considered and such insights help with the targeting of policies to particular groups of land managers, so that they align their motivations and operations. Additionally, if the right land managers are not targeted, there might be adverse selection from paying land managers who may have adopted the practice regardless of the payment (Canessa et al., 2024; Wunder et al., 2020), often referred to as self-selection bias.

The remainder of the paper includes an overview of agriculture in Ireland and the Netherlands, existing AES and attitudes towards these schemes in both countries, in Section 2. Section 3 describes the data and methodology. The results of our research are discussed in Section 4 and the implications of the findings are outlined in Section 5.

## Background

### Case Studies

Ireland and the Netherlands were chosen as case studies as they are EU Member States with pressing environmental issues stemming from the agricultural sector. Both countries are considerable net exporters of agricultural produce (CSO 2023; Statistica 2023), both have experienced rapid growth in the dairy sector following the EU milk quota abolition in 2015 (Läpple et al. 2021), and both have intensive livestock farming. The Netherlands has the highest and Ireland has the sixth highest livestock density in the EU (Eurostat 2023a), leading to pressure in both countries to reduce negative externalities such as declining biodiversity, water quality, and greenhouse gas emissions.

Despite these similarities, there are some interesting differences that make these two countries appropriate case studies. The countries differ in that the livestock production system in Ireland is predominately from grass-fed livestock (DECC 2022), with the dairy sector in the Netherlands relying on (imported and locally produced) feed, as well as grazing (Läpple and Sirr 2019). The Netherlands produces mainly cereals, feed crops and potatoes (Gov. of the Netherlands 2023). Also, the land markets differ significantly with the land rental market in the Netherlands being much more active than in Ireland with 39% of land rented in 2018, compared to 19% in Ireland (European Commission, 2021, p.51). Finally, as shown in the data section of this paper, the countries also differ in that AES are much more common in the Netherlands.

### Agri-Environmental Schemes in Europe

AES are the main mechanisms through which land managers are financially rewarded for operating in an environmentally friendly manner, above that required for the Basic Premium Scheme (Teagasc.ie 2022). As previously mentioned, reward for the adoption of AES has traditionally been ‘action-oriented’ payments with renumeration based on an action and not the outcome (Olivieri et al. 2021). This focus has been, partly, on providing compensation to cover the cost to the land manager (including expenses and income foregone) of adopting a particular agri-environmental practice. In 2013, 26% of the utilised agricultural area of EU countries was under an AES (Eurostat 2023b). While this seems favourable from an environmental perspective, the results of land managers’ actions are not measurable from this figure. Burton and Schwarz (2013) discussed scenarios where action-based contracts have failed to provide environmental benefits due to the entry of low quality land into the schemes and a possible focus of land managers on ease of management rather than ecological benefits. In addition, Mack et al. (2020) found from a study of Swiss farms that adverse selection in AES arose because some farm types could implement the required actions with little change to their practices.

Encouraging farmers to instead focus on the results of their actions may be more effective in increasing environmental benefits and this is reflected by growing interest from the European Commission. For example, the EU Biodiversity Strategy 2030 provides a governance framework to improve knowledge and financing, and to ensure better implementation and tracking of biodiversity improvements (European Commission 2024). Results-based payments are already being applied across Europe but predominantly on small scales (Chaplin et al. 2021) and examples of environmental improvements include the findings of Sidemo-Holm et al. (2018) which show that farmers adopt fewer but more effective pollution abatement measures when payment is based on environmental results. In addition, Franks (2011) argues that collective-action contracts can have high levels of ecological effectiveness. Therefore, we outline four innovative contract types that have the potential to foster environmentally friendly farm management at larger scales: results-based, collective action, value chain and land tenure contracts.

**Results-based contracts** provide land managers with payments that are based on the performance of their practices. For example, if the environmental goal is to increase the number of a bird species in an area, payments will be based on the number and types of birds identified over a period of time. An advantage of these contracts is that they place a greater emphasis on environmental outcomes than action-based contracts. As land managers are often free to choose their own measures, local knowledge and environmental learning are utilised (Uthes and Matzdorf 2013), which can aid the applicability of this contract type to a wide range of farms. Unpredictable external factors such as weather and market conditions can discourage land managers from taking up these contracts as they directly affect the results of land managers’ actions and, in turn, the payment (Olivieri et al. 2021). However, Lapierre et al. (2023) find that there are means to reduce external uncertainty from weather shocks in AES, such as offering land managers the option to suspend the conditions of the contract for one year, of their choice, while still receiving the payment for the full duration of a multi-year contract.

An example of an Irish AES where land managers enrolled in results-based contracts is The Burren Programme. This scheme sought to protect biodiversity in the Burren in West Ireland which is an UNESCO Geopark area of exposed limestone. Five-year environmental targets and action plans were agreed between farm advisors and land managers (CONSOLE 2021), with payments being dependent on land managers implementing plans and performing according to a 10-point evidence-based scoring system. The average score from the 147 participating farms increased from 6.61 in 2010 to 7.43 in 2019 (The Burren Programme 2024) and the success of this scheme is attributed to local leadership, high levels of local engagement and farm assessments being scientifically based (CONSOLE 2021).

**Collective contracts** require land managers to become members of a group which applies jointly for compensation to implement environmentally beneficial activities. The payments are then awarded based on the group’s performance. The Netherlands is the only EU Member State to have introduced a collective AES on a national level. The benefits of the collective approach are that it allows for management on landscape scale. This way, greater ecological effectiveness is achieved as delivery of specific public goods (e.g., biodiversity) can benefit from the larger scale and linkages (Prager 2015). Franks (2011) also argues that these contracts can have high levels of ecological effectiveness and they note that moral hazard^3^ is unlikely due to land managers wishing to maintain their reputation. However, it can be difficult to determine the optimum group size and to eliminate asymmetric information. Another problem could be mistrust among farmers, especially if they are dependent on each other to receive the payment, since farming is considered an individual rather than a collective endeavour (Riley et al. 2018; Šumrada et al. (2022)). The applicability of this contract type is, therefore, dependent on the relations of neighbouring land managers. For example, Villamayor-Tomas et al. (2021) find that the majority of surveyed Swiss farmers have low expectations about the willingness of their neighbours to act collectively in an AES, which creates an immediate barrier.

**Value chain contracts** involve land managers providing environmental benefits connected to the production of selected products. This is rewarded by the market, mainly through a premium price or by the right to use a label. For example, FrieslandCampina, the largest dairy cooperative in the Netherlands, developed the “On the way to planet proof” label^4^ to highlight the positive environmental aspects of their products. Approximately 700 farmers participated in this label in 2019 and, as per 2020 figures, these farmers received a premium of €0.02 per litre (Vermunt et al. 2022). Aside from encouraging farmers to adopt positive environmental practices, including greenhouse gas emissions reduction, increasing on-farm circularity and establishment of small landscape elements, an advantage of this type of contract is that it could provide indefinite additional income for farmers. However, there may be concerns that the higher price will not be transferred to the land manager. In addition, such contracts require the interest of commercial entities, which land managers may feel they have little connection to, without the help of an intermediary such as a farm advisor. Olivieri et al. (2021) describe the current literature on both value chain and tenure contracts as scarce, and it is the aim of our research to contribute to this area.

**Land tenure contracts** mean that a landowner accepts a lower lease payment than under usual land tenure agreements, to compensate land managers for their additional efforts to protect the environment. A landowner contractually requires the tenant to comply with certain management requirements, like reduced use of pesticides. These contracts ensure that, in this example, their soil quality is improved, and they may give landowners confidence that their land is being satisfactorily managed. However, potential challenges with this contract type can arise concerning agreement on the measurement of the success of the tenant’s actions. An active rental market is also required for land managers to feel that this contract type could be applicable to them.

The EU CAP Strategic Plans for 2023-27 for each country are currently operational and both results-based payments and a focus on collective action are key elements of the Plans for environmental improvements in Ireland (DAFM 2022). In addition to the four pure contract types discussed, innovative AES can consist of hybrid contracts that combine design and governance characteristics from more than one contract type (Bredemeier et al. 2022). For example, in Agrarische Natuurvereniging Oost-Groningen and Natuurrijk Limburg in the Netherlands, the province established a collective agreement with a cooperative which entered legally binding contracts with each individual farmer (Bredemeier et al. 2022).

Our study focuses on the characteristics of the afore-mentioned four pure contract types to determine what specific elements of a contract type are considered to be understandable, applicable and economically beneficial.

### Sectoral Challenges in Ireland and the Netherlands

Ireland has the greatest percentage of land dedicated to agriculture in the EU, at 72% (Eurostat 2022), and 54% of the total land area of the Netherlands is used for agriculture (StatLine, 2023). Although average farm incomes in both countries are often above national average incomes, in Ireland in 2021, 27% of farms were classified as economically vulnerable^5^ (Dillon et al. 2022) and at least 20% of Dutch farms report an income below the poverty standard (European Commission 2020, p.9). This highlights that some farms face considerable financial concerns as well as environmental challenges.

Ireland is committed to achieving a 51% reduction in greenhouse gas emissions from 2021 to 2030 and net-zero emissions by no later than 2050, with requirements to achieve these objectives set out in legislation (DECC 2022). Agriculture produced 33% of greenhouse gas emissions in Ireland in 2021 (DECC, 2022), and much of this can be attributed to Ireland’s large livestock sector (DECC 2021).

The importance of the livestock sector to agriculture in Ireland is highlighted by the fact that 91% of farms were specialist livestock farms in 2022 (Dillon et al., 2023). More than 80% of agriculture related greenhouse gas emissions in Ireland are directly attributed to livestock numbers and the management of the manures they produce. 12% is linked to chemical fertilisers, and the remaining 8% from fuel combustion and carbon dioxide from lime usage (DECC 2021). The Environmental Protection Agency notes that agriculture also significantly contributes to the decline in water quality nationally, with other sectors also contributing to this trend. The agriculture sector is also responsible for over 99% of national ammonia emissions (DECC 2021).

Environmental challenges in the Dutch agricultural sector relate to scale enlargement and the relatively intensive way of farming, which was exacerbated by the abolition of the EU milk quota in 2015. Environmental concerns include nitrogen deposition levels, greenhouse gas emissions and biodiversity loss. According to 2016 figures, nitrogen deposition is considered too high to ensure biodiversity protection in 70% of nature areas and approximately 40% of the nitrogen deposition originates from agriculture (European Commission 2020). Between 1940 and 2017, butterfly abundance in grasslands decreased by 80% (Van Strien et al. 2019) and Buijs et al. (2019) note that there are serious concerns about further declines in AECPG, with there being widespread support for contracts that will protect them.

While the Dutch economy as a whole has a greenhouse gas emission intensity (amount of greenhouse gas emissions per € added value) of 0.3 kg CO_2_ equivalents per € added value, the greenhouse gas emission intensity of the agricultural sector in 2018 was eight times as high, with 2.5 kg CO_2_ equivalents. Greenhouse gas emissions from agriculture decreased between 1990 and 2003 by 26%, but emission reductions have since slowed. Between 2020 and 2021, greenhouse gas emissions from agriculture declined by 2% in the Netherlands. This is more than twice the reduction of the EU agriculture sector in that period (Eurostat 2023c).

### Existing Agri-Environmental Schemes in Ireland

Irish AES have evolved since 1994 with the first being the Rural Environment Protection Scheme (REPS) 1, 2, 3 and 4 schemes. The schemes paid land managers for undertaking measures on a per hectare basis and this led to farm size being a strong determining factor in a farmer’s decision to participate (Hynes and Garvey 2009). The Agri-Environment Options Scheme (AEOS) replaced REPS in 2010 and it differed from REPS in that payments applied to individual measures at set rates, rather than across the whole farm (Cullen et al. 2021). The Green Low-Carbon Scheme (GLAS) replaced AEOS in 2015 and it involved the further targeting of funds to achieve greater scheme results (Cullen et al. 2021). It also included the targeting of areas with existing environmental need or value, in an attempt to address the lack of additionality which was identified as a problem in previous schemes (Cullen et al. 2021). All of these have now concluded and a new agri-environmental climate measure called the Agri-Climate Rural Environment Scheme (ACRES) was introduced in January 2023 which, like the previously discussed measures, is co-funded by the Irish Government and the European Agricultural Fund for Rural Development of the EU. It is part of Ireland’s CAP Strategic Plan 2023-27 (DAFM 2022). The objective of ACRES is to address biodiversity decline while also providing income support. There are two entry points, a nationwide approach offering a range of measures for individual land managers and a separate approach for high priority geographical areas where a level of co-operation is required amongst participants (DAFM 2022).

### Existing Agri-Environmental Schemes in the Netherlands

In the 1970s, it was observed that, following World War II, developments such as land consolidation, nature, landscape and agriculture were no longer automatically connected in the Netherlands (Kuindersma et al. 2018). In response, the first policy addressing nature conservation by agriculture was implemented. This policy had two tiers: 1. Converting designated agricultural lands to nature conservation areas (land sparing) and 2. On-farm management practices were implemented to enhance nature (land sharing) (Green et al. 2005), specifically focussing on meadow birds. Following limited success, this policy was revised in 1990. The land sharing approach failed, due to an aversion towards the integration of nature management (Kuindersma et al. 2018). A new policy was constructed with the primary aim to halt biodiversity loss and fragmentation of nature by creating an ecological core network. Then, at the beginning of 2000, the attitude of the Dutch government moved towards a shared responsibility with society as a whole. Simultaneously, EU policy had a growing influence on the Dutch national nature policy (Runhaar 2017) and the concept of (regaining) multifunctionality was gaining interest, increasing the willingness to engage in agricultural nature management. Meanwhile, scale enlargement and intensification kept growing.

Together, this led to the current agri-environmental measures in the Netherlands which are implemented through collective contracts. Dutch land managers who choose to participate in AES must become a member of an agricultural collective, which is responsible for the individual contracting, and coordinates measures on a landscape scale. The agricultural collectives in the Netherlands are legal entities that are responsible for carrying out the AES, as the ultimate beneficiaries of the subsidy. Besides implementing the AES, the collectives provide other benefits such as knowledge exchange among their members to promote collaboration with other actors such as water authorities, nature conservation groups, and industry partners (Barghusen et al. 2021; Terwan et al. 2016). Many of the local cooperatives are responsible for creating and maintaining landscape elements (CONSOLE 2020) or meadow bird protection. Boonstra et al. (2021) concluded that the collective approach ensures greater involvement of land managers in management and more flexibility to adapt measures to conditions in the field in the Netherlands. They also note that some ecological conditions have slightly improved. However, meadow bird protection is still falling short, and improvements can be made, for example, by lowering the administrative burden and through more knowledge sharing (Boonstra et al. 2021).

In terms of value chain contracts, several exist in the Netherlands, for example, between supermarkets and their suppliers who are arable land managers, and between dairy processing firms and their supplying land managers. This results in dairy land managers receiving a premium on the milk price when, for example, they increase animal welfare or protect biodiversity in their meadows (Royal Aware 2015). Besides private companies, non-governmental organisations are involved in such contracts. They can, for example, issue a label for land managers who protect agrobiodiversity (Vogelbescherming 2020). Much of the information on environmental land tenure contracts in the Netherlands is anecdotal. Four large investment companies own 8.6% of the leased land (CBS 2021), and one of them (ASR Real Estate) offers 5 to 10% discount on land lease upon the implementation of specific environmental measures related to soil quality and biodiversity, and the commitment to a business plan with farm-specific sustainability measures (ASR Real Estate 2024).

### Factors that Affect the Adoption of AES and Innovative Contracts

#### Understandability, Applicability, Perceived Economic Benefits and AES adoption

The objective of this research is to understand whether land managers agree that results-based, collective action, value chain and tenure contracts for the delivery of AECPG are understandable, applicable to their farm, and economically beneficial. Existing literature shows that these three traits are important for encouraging uptake. For example, Dessart et al. (2019) highlight that a clear prerequisite for adopting more sustainable practices is understanding the practices or policy-supported schemes that exist. Pavlis et al. (2016) found, in a study of five EU countries, including the Netherlands, that farmers most often noted a lack of knowledge about voluntary agri-environmental schemes as the reason they did not participate in them.

Canessa et al. (2024) argue that alignment is the first aspect considered by farmers when deciding whether to enter an agri-environmental measure or not. This means that farmers are assessing the contract measure’s practical relevance (Whitten et al. 2013) or its compatibility with their values, past experiences, and needs (Rogers 2003). As recommended by Whitten et al. (2013), the AES should be suitable for land managers’ production outcomes to be considered aligned (Whitten et al., 2013) and to increase adoption. Kelemen et al. (2023) studied the same four innovative contract types as this study by conducting a Policy Delphi study with an expert panel from 15 European countries. They find that collective contracts are the least suited to existing institutions, and the social and cultural context, while value chain contracts are the most suited to these contexts.

There is a positive link between the economic benefits of AES, which accrue to the land manager, and adoption rates. For example, Wilson and Hart (2000) studied EU farmers’ motivations to participate in voluntary AES and found that economic considerations were the primary driver for farmers to participate in AES (79% gave financial reasons; 64% stated a secure source of income), followed by reasons that schemes suited current farm-management plans (‘goodness of fit’) (50%). In addition, Peerlings and Polman (2009), found from a study of five European countries^6^, including the Netherlands, that an increase in contract payments increases the amount of land within a farm that is allocated to agri-environmental contracts.

According to a study of twelve European countries by Bradfield et al. (2024), annual compensation, guaranteed sales, and higher payments for better results are the main contributors to land managers’ willingness to adopt environmental contracts. Vermunt et al. (2022) identified five key mechanisms that hinder the adoption of nature-inclusive agriculture in the Dutch dairy sector. They are insufficient economic incentives for farmers, limited action perspective of many dairy farmers in the Netherlands^7^, lack of a concrete and shared vision for natural inclusive agriculture, insufficient specific and integral knowledge, and regime resistance. In addition, Pavlis et al. (2016) find that economic gains considerably influence AES adoption in the Netherlands and personal satisfaction^8^ is only considered important by just over half of their Dutch sample. However, the literature does not identify the specific factors associated with the perceived economic benefits of AES for land managers, which is the focus of our research, with the added novelty of applying it to innovative contract types.

#### Land Holding Characteristics and AES Adoption

Due to the heterogeneity of land managers, there can be uncertainty regarding which farmer groups are most likely to participate in agri-environmental contracts (Niskanen et al. 2021). The characteristics of the farm’s production system, such as degree of specialisation and type of production practices in place, determine farmer availability for specific management actions, such as engagement with AES (Mozzato et al. 2018). Therefore, knowledge of the differences between farmer groups in their reactions to agri-environmental contract design is important for policy design, as incentives may need to be tailored for different groups. For example, Cullen et al. (2020) found that sheep farmers were the most likely to have participated in AES in Ireland, with more intensive systems such as dairy, tillage and intensive beef production being approximately 50% less likely to participate. Paulus et al. (2022) found that permanent grassland was more likely than arable land to be under an AES due to lower levels of intensity in Germany, and intensively farmed grasslands discouraged re-enrolment in AES in Italy (Gatto et al. 2019). These findings highlight that the relationship between farming systems and AES enrolment is dependent on the farming intensity level.

Farm sizes have also been found to influence AES adoption. Increasing farm size is often associated with an increased likelihood of AES adoption or continuation (Cullen et al. 2021; Gatto et al. 2019; McGurk et al. 2020). For example, Cullen et al. (2020) found that adoption of AES in Ireland is most likely on farms of 100 to 150 hectares. These are relatively large farms as only 8% of farms in that study were of that size or larger. Similarly, Paulus et al., (2022) found a positive relationship in Germany but they note that the opposite is true for Italy, as studied by Capitanio et al. (2011). The reasoning provided is that average farm sizes vary significantly in different countries.

Defrancesco et al. (2008) found that the portion of farm income relative to total household income has a negative effect on AES adoption in Italy and Barreiro-Hurle et al. (2010) found that farm income has a negative impact on farmers’ willingness to participate in an AES in Spain. This may be due to the financial risk of adopting less intensive farming practices to adhere to these schemes (Lastra-Bravo et al., 2015; Wossink and van Wenum 2003). However, Cullen et al. (2021) showed that increasing farm incomes have a positive impact on adoption in Ireland which suggests that the opportunity cost of AES enrolment is low. Cullen et al. (2021) found that a €1000 fall in income decreases the likelihood of farmers’ adoption by 1–2 percent in Ireland. The relationship between age and AES adoption is unclear because Hynes and Garvey (2009) and Cullen et al. (2021) found that age has a positive association with AES adoption, but McGurk et al. (2020) found this to be a positive non-linear relationship.

McGurk et al. (2020) found that participation in the land rental market has a negative impact on AES adoption in Ireland. This may be due to uncertainty about the future or because an agreement between landlords and tenants is required before entering an AES contract (Lastra-Bravo et al. 2015; Wilson and Hart 2000), and land rental rates in Ireland are low relative to other European countries. Defrancesco et al. (2008) found that land tenure had a positive effect on farmers’ willingness to engage with grassland conservation in Italy. Ruto and Garrod (2009) found, from a study of ten EU countries, that farmers who rent a large portion of their land had a preference for short-term contracts, noting that it is unclear whether this finding reflects uncertainties over the duration of tenancies or the influence of landlords who might not wish for their land to be tied into a long-term contract.

Farmers’ experiences with past AES and other environmentally friendly farming practices have been shown, in numerous studies, to have positive effects on farmers’ willingness to adopt a new AES (Cullen et al. 2021; Defrancesco et al. 2008; Hynes and Garvey 2009; Wilson and Hart 2000), which suggests that land managers are satisfied with such schemes. Gatto et al. (2019) found that training and skills development were positively associated with farmers’ AES continuation in Italy, which is to be expected.

Existing research has also explored the motivations for adopting innovative contracts. For example, Niskanen et al. (2021) surveyed Finnish farmers regarding their preferences to accept results-based agri-environmental policies compared to the existing practice-based approach. Nearly half of the participants were noted as requesting high levels of financial compensation and they were typically young. They did not support extensification and they had large farms. Another large group (24%) favoured high compensation but with strong resistance to policy change. They were typically crop producers with small to medium size farms and they did not support extensification. Some farmers (19%) supported policy reform with moderate financial requests. The remaining farmers, who tended to be older with small farm sizes, supported extensification. They typically had livestock farms and engaged in organic production.

Barghusen et al. (2021) surveyed farmers in the Netherlands and they found that farmers’ motivations to participate in collective AES are partly driven by economic considerations. Personal norms concerning environmental measures such as problem awareness, perceived responsibility, and a feeling of collective efficacy are found to be equally important. Thiermann et al. (2023) analysed Dutch farmers’ willingness to accept a hypothetical, hybrid results-based and collective action scheme for the protection of meadow birds. They found that farmers who were younger, less educated, and had a large farm size and a strong financial position were the most likely to accept the scheme. They were also less likely to have dairy farming as their main occupation.

Bradfield et al. (2024) studied results-based, collective action, value chain and land tenure contracts and found that results-based contracts are considered to be understandable, applicable and economically beneficial by most surveyed respondents across twelve European countries^9^, with these figures being particularly high in Ireland. Kelemen et al. (2023) found that some of the main barriers to adoption of these contracts were due to gaps in knowledge, economic, including budgetary constraints, higher transaction costs due to monitoring and enforcement, increased risks and uncertainty. However, the characteristics of land managers in Ireland and the Netherlands who perceive these innovative contracts to be economically beneficial have not been studied.

## Data and Methodology

### Survey Data

Survey data were collected as part of an EU Horizon 2020 funded project, CONSOLE, which analysed contract solutions for the effective and lasting delivery of AECPG. The surveys^10^, which contained identical core questions in all project partner countries, were distributed to Irish and Dutch land managers online by research marketing agencies between January and July of 2021.^11^ Agencies in both countries contacted land managers from differing farming systems to ensure the data captured varying perspectives. The focus was on land-based agriculture, defined as farms whose production relies entirely or predominantly on the land’s production capacity. Therefore, zero grazing farms, greenhouses and poultry farms are not included in the study. The land managers were not contacted based on their experience of AES in any format. The target sample size of the surveys was a minimum of 100 land managers per country to ensure that there is a variety of farms in the sample. The final sample included 210 land manager surveys completed in Ireland and 201 completed in the Netherlands.

The surveys included questions on land holdings and land managers’ characteristics, which included factors that are known to influence farmers’ decisions, e.g. age, farm size, education, farm orientation/type, et cetera. The focus of our analysis includes three questions in which land managers were asked ‘Do you agree or disagree with the following statements?’ after they received an explanation of the contract type. The explanations are provided in the Appendix and the statements are:

- The contract type is easy to understand.
- The contract type is applicable to my land.
- The contract type is economically beneficial for me.

**Fig. 1:**
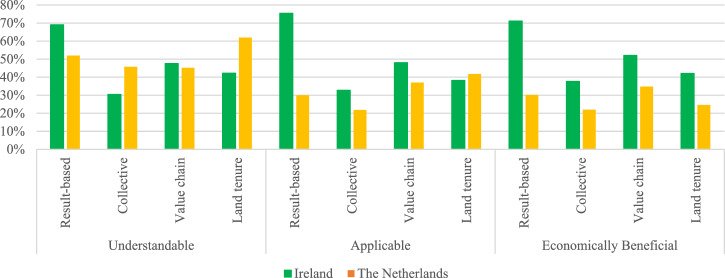
The Percentage of Land Managers Who Agree

The respondents selected one of five options on a Likert scale: strongly agree, agree, neutral, disagree and strongly disagree. As the statements are broad, they call on respondents to consider all aspects of their land and all characteristics of a contract when stating a response. Figure 1, which is provided in the Results and Discussion section, graphs the percentage of land managers who agree with a statement.

### Data

#### Probit Models

Probit models were used to assess the factors influencing a binary outcome which, in this case, is whether land managers agree^12^ that a particular type of contract is economically beneficial or not (Greene 1993; Wossink and van Wenum 2003). The model is as follows;

1 $${a}_{i}={B}_{0}+{\sum }_{j=1}{B}_{J}{x}_{{ij}}+{u}_{i}$$

Where *B* is the constant, ${x}_{{ij}}$ denotes a set of explanatory variables *j* for land manager *I* and ${u}_{i}$ is the error term.

*a*_*i*_ = 1, if a land manager agrees that a contract is economically beneficial.

*a*_*i*_ = 0, otherwise.

Respondents were asked about the contract types in isolation and, therefore, it is not possible for us to assess land managers’ preferences for them as alternatives to each other. Canessa et al. (2024) find that Probit and Logit models are the predominant methodologies used by researchers of the adoption of agri-environmental climate measures. As our research examines a binary outcome, agreement with economic benefits, a Probit model is appropriate. Average marginal effects are reported and variance inflation factor tests confirm that multi-collinearity is not evident in the models. The literature review, as presented above, guided the selection of explanatory variables from the available dataset. ‘Formal agricultural/forestry training’ is included as an explanatory variable in the Probit models assessing data from Ireland. However, this variable is omitted from the Probit models for the Netherlands due to it being collinear with other variables in the data. Land managers’ contact with advisory services was considered for inclusion in the Probit models. However, 100% and 94% of respondents engage with these services in the Netherlands and Ireland, respectively. This means that its inclusion would add no value to the model and its results. Pearson chi-square tests confirm goodness-of-fit. Tables 3 and 4 provide results of the Probit models.

## Results and Discussion

### Land Holding Characteristics

Tables 1, 2 provide summary statistics of the land managers in both countries and the data of the survey respondents can be compared to nationally representative data^13^. The Farm Accountancy Data Network (2021) data on farm types, farm sizes and rented land, and data collected by Eurostat (2021a) on formal agricultural training can be directly compared with the sample of this study. However, Eurostat (2021b) collected data on farm holders’ age categories that differ from those used in our survey. For comparison purposes, we used data collected by Eurostat (2016) on farm holders who describe their main economic activity as being derived from their farm as a proxy variable for farm income being more than 50% of total income. To the best of the authors’ knowledge, data on the current use of innovative agri-environmental contracts are not currently collected on an official level that is representative of the European Union or its Member States.

**Table 1:** Descriptive Statistics of sampled land managers and comparison with nationally representative data (Proportions).

	Ireland		The Netherlands	
	Sample	Nationally Representative Data	Sample	Nationally Representative Data
*Farm system*				
Dairy	0.55	0.17	0.56	0.33
Cereals and Crops	0.01	0.03	0.34	0.36
Mixed Systems	0.01	0.02	0.04	0.02
Cattle	0.21	0.60	–	0.11
Other/Mixed Livestock	0.21	0.18	0.01	0.15
Forestry	0.01	–	–	–
Fruit/Vineyards	–	–	0.04	0.03
Other	–		0.01	–
*Agri./Forestry education completed*	0.82	0.54	0.88	0.90
*Farm Income* > *50% of Total Income*	0.66	0.52	0.87	0.20
*Age of land manager*				
Age 18–30	0.06		0.04	
Age 31–50	0.53		0.30	
Age 51–70	0.39		0.51	
Age 71 and older	0.02		0.15	
<25 Years		0.01		0.01
25–29 Years		0.08		0.05
30–34 Years		0.07		0.06
35–44 Years		0.10		0.09
45–54 Years		0.27		0.36
55–64 Years		0.24		0.32
65 Years or Older		0.23		0.10

Descriptive Statistics of sampled land managers and comparison with nationally representative data (Means)				
	Ireland		The Netherlands	
	Sample Mean (Std. Dev.)	Nationally Representative Mean	Sample Mean (Std. Dev.)	Nationally Representative Mean
*Farm Size (hectares)*	76.04 (44.64)	46.2	98.86 (424.27)	40.7
*Rented to Owned Land Ratio*	0.54 (0.75)	0.21	3.53 (26.09)	0.44

**Table 2:** Descriptive Statistics of sampled land managers—Proportions

Ireland		Ireland	The Netherlands
*Experience with contracts*			
Results-Based Contract	Currently Using	0.30	0.26
	Never Used	0.66	0.67
	Previously Used	0.04	0.07
Collective Contract	Currently Using	0.17	0.39
	Never Used	0.81	0.60
	Previously Used	0.02	0.01
Value-Chain Contract	Currently Using	0.16	0.34
	Never Used	0.82	0.64
	Previously Used	0.02	0.02
Tenure Contract	Currently Using	0.03	0.19
	Never Used	0.96	0.78
	Previously Used	0.01	0.03
*Preferred contract length*			
	<1 Year	0.05	0.21
	1–5 Years	0.72	0.58
	5–10 Years	0.23	0.21

While our sample contains a disproportionate number of dairy farms, the sample does reflect the importance of the livestock sector to Irish and Dutch farming. Our sample also reflects the high education of farm holders in both countries. Our sample contains large farms with a high portion of rented land.

As noted in Table 2, 30% of surveyed land managers in Ireland are currently using results-based contracts, 17% are using collective action, 16% are using value chain and 3% are using land tenure contracts. In the Netherlands, 26% of surveyed land managers are currently using results-based contracts, 39% are using collective action, 34% are using value chain and 19% are using land tenure contracts. Medium length contracts, of 1–5 years, are the preferred duration in both countries.

### Perceptions of Innovative Contract Designs

Figure 1 shows the percentage of land managers who agree^14^ with the following statements:

- The contract type is easy to understand.
- The contract type is applicable to my land.
- The contract type is economically beneficial for me.

#### Perceptions of Results-Based Contracts

Figure 1 shows that the greatest portion of land managers in Ireland (69%) understand results-based contracts, which is a higher percentage than in the Netherlands (52%). In Ireland, 75% of land managers agree that results-based contracts are applicable to their farm compared to 30% in the Netherlands. The percentage of land managers who perceive results-based contracts to be economically beneficial in Ireland (71%) is also much higher than in the Netherlands (30%). This may be explained by the fact that results-based contracts are relatively common in Ireland, compared to the three other contract types, and land managers may have seen them in use and experienced their benefits. This follows the findings of Wilson and Hart (2000) who noted that non-familiarity with AES can increase the likelihood of farmers being unable to agree with their benefits.

#### Perceptions of Collective-Action Contracts

Collective contracts are considered understandable (30%), applicable (33%) and economically beneficial (38%) by the fewest land managers in Ireland. As farms in Ireland are predominately family owned, this may explain a hesitancy towards collective action because external influences on management decisions would not be common. In contrast, and as previously mentioned, collective contracts are an essential part of AES in the Netherlands which makes them more common and this has resulted in a higher percentage of Dutch land managers understanding them (46% compared to 30% in Ireland). However, applicability and perceived economic benefits of these contracts are low in both countries, which follows the findings of Kelemen et al. (2023). A reasoning for these findings may be that compensation has been low, as discussed by Westerink et al. (2018) and Beldman et al. (2019). It might also be linked to concerns over monitoring and enforcement, and risk and uncertainty, as discussed by Kelemen et al. (2023).

#### Perceptions of Value Chain Contracts

Value chain contracts are understood by 48% of surveyed land managers in Ireland and 45% of surveyed Dutch land managers. Agreement with the applicability and economic benefits of these contracts is also higher in Ireland, despite current use of these contracts type being very low. Value chain contracts are considered to be economically beneficial by 52% of surveyed land managers in Ireland compared to 35% in the Netherlands. These findings suggest that there may be demand for these contracts in Ireland that is not currently being satisfied. Compared to the other three studied contract types, more land managers in the Netherlands feel that value chain contracts would be economically beneficial for them, which follows the findings of Kelemen et al. (2023). The reason for this may be familiarity, as the Dutch dairy sector is organised by a few large dairy processing cooperatives, which already have value chain contracts in place (Vermunt et al., 2022).

#### Perceptions of Land Tenure Contracts

Land tenure contracts are understood by 42% of surveyed land managers in Ireland and 62% of land managers in the Netherlands. Applicability rates are also higher in the Netherlands. As previously noted, Ireland has a low level of land rental which is typically on short-term agreements (Bradfield et al. 2023) and the fact that 43% of land managers feel that a land tenure contract could be applicable to their farm may suggest a desire for the opportunity to enter such contracts. Of the four studied contract types, land tenure contracts are understood and deemed applicable by the greatest percentage of surveyed Dutch land managers which may be explained by their land rental market which is much more active than Ireland’s. However, a low percentage of Dutch land manager (25%) agree that they are economically beneficial.

It is worth highlighting that a lower percentage of land managers in the Netherlands than Ireland perceive the four contract types to be economically beneficial and we explore the characteristics of these land managers and their farms in the following section.

### Explanatory Factors of Perceived Economic Benefits

#### Ireland

It is clear from the existing literature that perceived economic benefits are a deciding factor for land managers when entering innovative agri-environmental contracts. For that reason, the characteristics of land managers who agree that a contract type is economically beneficial are of interest and are analysed here using Probit Models. Table 3 displays the results of Probit models using Irish data. 177 observations are analysed and robust standard errors are reported.

**Table 3:** Probit Models for perceiving contracts as economically beneficial for Ireland (average marginal effects)

	Results-Based		Collective		Value Chain		Land Tenure	
	Coef.	Std. Err.	Coef.	Std. Err.	Coef.	Std. Err.	Coef.	Std. Err.
Dairy (ref.)								
Cattle Rearing	0.07	0.11	−0.03	0.12	−0.11	0.12	−0.19*	0.11
Mixed Livestock	0.16	0.10	0.00	0.10	−0.10	0.11	−0.02	0.10
Mixed Systems	–	–	0.29	0.26	−0.25	0.33	−0.24	0.25
Farm Size	0.00	0.00	0.00	0.00	−0.00	0.00	0.01*	0.00
Farm Size (sq.)	−0.00	0.00	−0.00	0.00	−0.00	0.00	−0.00**	0.00
No Agri./For. Training (ref.)								
Agri./Forestry Training	−0.12	0.11	0.10	0.10	0.05	0.11	−0.01	0.11
18–30 Years Old (ref.)								
31–50 Years Old	−0.24***	0.07	−0.21	0.15	−0.20	0.15	−0.07	0.16
51–70 Years Old	−0.27***	0.08	0.21	0.16	−0.26	0.15	−0.22	0.16
71 Years or Older	–	–	−0.09	0.37	–	–	–	–
Contract Term <1 Yr (ref.)								
Contract Term 1-5 Years	0.02	0.16	0.15	0.12	−0.16	0.16	−0.20*	0.14
Contract Term 5–10 Years	0.16	0.16	0.35***	0.13	0.02	0.17	−0.10	0.15
Rented/Owned Land	0.16*	0.09	−0.09	0.05	0.26*	0.16	0.42***	0.10
Rented/Owned Land (sq.)	−0.05**	0.02	0.00	0.02	−0.08	0.07	−0.09***	0.03
Farm Y > 50 Total Y	0.13	0.08	−0.03	0.09	0.13	0.09	−0.09	0.09
Contract Usage—Never (ref.)								
Contract Usage—Current	0.12	0.09	−0.11	0.09	0.09	0.09	0.07	0.08
Contract Usage—Previous	−0.26*	0.15	0.10	0.17	−0.06	0.17	0.18	0.16

*** p < 0.01, ** p < 0.05, * p < 0.1

With regards to results-based contracts in Ireland, the older the farmer, the less likely they are to agree that results-based contract are economically beneficial for them. This contradicts the findings of Hynes and Garvey (2009) and Cullen et al. (2021) who assessed adoption of AES in Ireland, without considering innovative contract designs.

Our findings somewhat reflect those of Mack et al. (2020), who note that young farmers have a higher portion of land under results-based contracts in Switzerland, as we find that young and/or renting land managers are the most likely to agree that results-based contracts are economically beneficial. As young land managers plan for the future and renting land managers occur a high opportunity cost to rent in land, they may share similar mindsets However, our findings contradict those of Niskanen et al. (2021) who studied the acceptance of results-based contracts in Finland. They found that a large cohort of young farmers with large farmers did not support results-based schemes with extensive farming practices.

We find a positive non-linear association between rented land and the likelihood that results-based contracts are perceived to be economically beneficial. It may be the case that results-based contracts provide renting land managers with a sense of control over their payments, when they have uncertainty over their land resources. This is supported by the fact that land rental agreements in Ireland are predominately conacre agreements and, therefore, often offer little security. Land managers who have never adopted a results-based contract are more likely than previous users to perceive this type of contract to be economically beneficial. Further research is required to determine if this finding is due to unfavourable experiences amongst previous users or a strong desire of non-users to engage in such contracts. Only 4% of survey respondents who have used this contract type no longer do so. The high retention rate may suggest an overall positive outcome to these contracts.

Regarding collective contracts in Ireland, a positive association exists between a long-term agreement (5 -10 years) and perceived economic benefits. As these contracts require collaboration and mutual understanding, land managers may feel that it can take time to maximise the benefits of this arrangement. Farms with a high portion of rented land are more likely to agree that value chain contracts are economically beneficial and further research may determine the reasoning for this.

For land tenure contracts, cattle rearing land managers are less likely to agree that this contract type is economically beneficial, when compared to dairy land managers. Dairy land managers in Ireland earn the highest average farm income (Dillon et al., 2022), making them the category with the greatest earning potential from renting additional land. Therefore, contract payments should take account of the heterogeneity of opportunity costs between farming systems, as also noted by Mack et al. (2020). Land managers with larger farms^15^ and a higher portion of rented land are more likely to agree that tenure-based contracts are economically beneficial which is intuitive given the direct benefits these contracts offer them. Land managers with a preference for medium term contracts are the least likely to agree that land tenure contracts are economically beneficial. This may be due to fact most land rental agreements are of a short-term nature, of 11 months, in Ireland. The Irish Government, since 2015, has been providing tax incentives for landowners to rent out their land on long term leases, with leases of 15 years or longer providing the greatest tax relief (Bradfield et al. 2023). This means that medium term contracts may not be seen as part of traditional land rental practices in Ireland or as being financially attractive, based on this policy. However, in reality, both medium and long-term leases are becoming more common (Bradfield et al. 2023) and this perception of medium leasing contracts being rare may change in time.

#### The Netherlands

Table 4 displays the results of Probit models using data from the Netherlands. 140 observations are analysed and robust standard errors are reported.

**Table 4:** Probit Models for perceiving contracts as economically beneficial for the Netherlands (average marginal effects)

	Results-Based		Collective		Value Chain		Tenure	
	Coef.	Std. Err.	Coef.	Std. Err.	Coef.	Std. Err.	Coef.	Std. Err.
Dairy (ref.)								
Cereals and Crops	−0.01	0.09	−0.24**	0.10	0.10	0.09	−0.18*	0.10
Fruit and Vineyards	0.22	0.22	−0.02	0.20	0.06	0.20	−0.36	0.24
Mixed Systems	−0.14	0.22	0.04	0.14	0.25	0.19	−0.10	0.16
Farm Size	0.00	0.00	0.00	0.00	−0.00	0.00	−0.00	0.00
Farm Size (sq.)	−0.00	0.00	0.00	0.00	0.00	0.00	0.01*	0.00
18–30 Years Old (ref.)								
31–50 Years Old	−0.22	0.25	0.03	0.23	−0.45**	0.21	−0.01	0.25
51–70 Years Old	−0.18	0.24	−0.09	0.22	−0.41**	0.20	−0.13	0.24
71 Years or Older	−0.33	0.25	−0.04	0.25	−0.52**	0.22	−0.24	0.25
<1 Year Contract (ref.)								
1–5 Years Contract	0.00	0.09	0.17**	0.07	−0.03	0.10	0.14	0.10
5–10 Years Contract	−0.03	0.12	0.12	0.09	0.12	0.13	0.13	0.13
Rented/Owned Land	−0.16	0.65	0.64	0.49	0.23	0.65	1.52**	0.71
Rented/Owned Land (sq.)	0.07	0.67	−0.40	0.49	−0.51	0.69	−1.21*	0.72
Farm Y > 50 Total Y	0.08	0.13	0.12	0.12	0.10	0.13	−0.10	0.12
Contract Usage – Never (ref.)								
Contract Usage – Current	0.27***	0.08	0.12	0.08	0.34***	0.08	0.16*	0.09
Contract Usage – Previous	0.04	0.15	−0.05	0.11	0.15	0.15	0.08	0.16

*** p < 0.01, ** p < 0.05, * p < 0.1

With regards to results-based contracts, the findings for the Netherlands show that land managers with results-based contracts currently in place are more likely to agree that they are economically beneficial, when compared to land managers who have never engaged with this contract type. This is an important finding because it shows that the practical experience of these contracts is positive. For collective contracts, cereal and crops land managers are less likely than dairy land managers to agree that collective contracts are economically beneficial, because this type of farming requires significant investment on the land and they may worry that it will not be rewarded or easily reversed. There is also a long history of dairy cooperatives in the Netherlands which may be driving land managers’ agreement that this form of management can be economically beneficial for such farmers. Land managers with a preference for medium term contracts are more likely to agree that collective contracts are economically beneficial, relative to those with a preference for a short-term contract. Medium term contracts may provide a balance of certainty and flexibility for both parties.

Land managers currently using value-chain contracts are more likely to agree that they are economically beneficial than those who have never used the contract type. Young land managers are the most likely to agree with the economic benefits of value chain contracts. For land tenure contracts, cereal and crop producers are less likely than dairy land managers to agree that this contract type would be economically beneficial for them. A positive, non-linear, association exists between land rental and agreement, and current users are the most likely to agree that they are economically beneficial.

## Implications and Conclusions

The objective of the research is to assess if land managers in Ireland and the Netherlands agree that results-based, collective action, value chain and land tenure contracts are understandable, applicable to their land, and (potentially) economically beneficial. Furthermore, we explore the characteristics of land managers who agree that these innovative contracts are, or would be, economically beneficial for them, which has not been researched in an Irish or Dutch context previously. Determining land managers’ understanding and perceptions of agri-environmental contracts is important for policymakers who wish to incentivise the provision of AECPG, which is a priority of the EU CAP.

We find that results-based contracts are considered to be understandable, applicable and economically beneficial for a large proportion of land managers in Ireland. In the Netherlands, land tenure contracts are understandable and applicable to the greatest percentage of land managers, and value-chain contracts are perceived to be economical beneficial by the greatest portion of land managers. These differing opinions across the two countries support the devolution of policy design to EU Member States, as different policy measures will be acceptable in different countries. The portion of land managers who agree that collective contracts are applicable and economically beneficial is low in both countries, despite this contract type having the potential to offer large scale ecological benefits through collaboration (Prager, 2015).

Based on the findings of this study for Ireland, there is potential scope for the encouragement of older farmers to engage with results-based schemes. This may require increased education, and evidence of the ecological and economic success of existing results-based contracts to change the mindset of some older farmers. It is important that this is a two-way conversation between land managers and policymakers to determine the ecological, structural, financial, cultural and operational barriers that are causing older farmers to disagree with the economic benefits of results-based contracts. It is also clear from our findings that collective contracts should be of 5–10 years to encourage their uptake in Ireland. This period should be long enough for environmental benefits to be achieved. The system of cooperatives used in the Netherlands to manage agri-environmental contracts could be considered for Ireland to encourage adoption as Ireland already has existing dairy processing cooperatives and the management of AECPG from these enterprises could be an initial step in the increased management of such contracts.

The results show that land managers in Ireland with a large portion of rented land perceive results-based, value chain and, unsurprisingly, land tenure contracts to be economically beneficial. If it is assumed that these land managers rent in land because they are otherwise constrained by their land resources, we can also assume that there are some non-renters who are not constrained by their land resources. They may have some land which is under-utilised from an economic perspective and, given the lower opportunity cost, it might be economically beneficial for them to enter this land into an agri-environmental contract. Therefore, it is important that farm advisors educate these farmers on the environmental and economic benefits they may gain from entering such land into these contracts. There are also challenges for increasing value chain contracts as they need to be attractive for parties in the supply chain, which is ultimately driven by consumer demand and a willingness to pay a higher premium for goods produced in an environmentally friendly manner.

Current users of results-based, value chain and land tenure contracts in the Netherlands are more likely to perceive these contract types as being economically beneficial than those who have never used the contract type. These ‘success stories’ should be widely communicated to encourage further adoption both in the Netherlands and in other countries. For example, given the relative hesitancy of adoption of these contracts in Ireland, Irish policymakers could learn from the successes of the Netherlands, despite the sectors differing in both countries. For example, Bazzan et al. (2023) found that trust building and social learning have been instrumental in the successful implementation of collective management schemes in the Netherlands, and learning about the practical ways to achieve both would be very useful. As noted by Zindler et al. (2023), greater integration of stakeholders in the policy-making process of AES is required.

In the Netherlands, enhanced efforts could be made to encourage older farmers to adopt value chain contracts or to encourage value chain actors to offer contracts to older farmers. Increased financial support, in this case higher premiums, education or the help of an intermediary agent to negotiate the contract may be beneficial. Collective contracts should be of a 1–5 year duration which is shorter than the recommendation for Ireland. As these contracts are more common in the Netherlands, this which may reduce mistrust between farmers or concerns over requiring a long period of time to recoup the benefits of such a contract. Alternatively, Dutch farmers may desire short contracts for increased flexibility.

In conclusion, policy could target particular groups of land managers, who are outlined in this study, to promote the economic benefits of innovative contract types as a means to encourage adoption. This conclusion should be considered while acknowledging the limitations of this study. For example, we did not account for explanatory factors beyond socio-economic demographics, such as the economic climate, existing policies, social norms et cetera. Additionally, our sample is not fully representative of the farming population in each country, as the survey was conducted on a non-probability sample. Nevertheless, the results provide a first indication of land managers’ perceptions of innovative contracts, and the comparison between two countries demonstrates the generalisability as well as the context specificness of potential contracts.

This topic would benefit from further qualitative studies that explore the opinions of land managers who have experience of using these contracts and, therefore, possess thorough understanding of their advantages and disadvantages. Comparing contract types as part of a choice experiment could yield valuable insights. Future research could also focus on specific farming systems to identify the contract features that provide the best environmental and economic benefits for those farmers. However, these findings remain useful for policymakers in determining their target market for each of these innovative contract types.

## Descriptions of innovative contract types provided to survey respondents

### Result-based contract

In a result-based contract, you receive a **payment only for the delivery of environmental or climate results**. You are **free in your decision about the management practices**, e.g. how to contribute to water protection, landscape improvement, biodiversity or to sequester carbon. Selected indicators and scoring systems to monitor environmental or climate results are often used, and they will be exactly defined in the contract. You have access to free advice or training when you participate in this contract and you can voluntarily engage in the monitoring activity.

### Contract with collective implementation

You become **a member of** a **group** of land managers (farmers or foresters) who **applies jointly for compensation in order to implement environmental or climate activities**, e.g. water protection, carbon sequestration, biodiversity or landscape improvement. A minimum number of group members (e.g. 5) from your region is required to **collaborate in order to get a payment**. The group members decide about the implementation and locating the measures, and the distribution of the payment. Within the group, peer land managers and advisors share knowledge and support the achievement of the environmental objectives.

### Contract along the value chain

As a producer, you are part of the value chain (producer, processor, retailer, distributor). You engage in a contract where you commit to deliver **environmental or climate benefits connected to the production of selected products**, e.g. by carrying out management measures which contribute to water protection, landscape improvement, biodiversity or carbon sequestration. Often these products get a special label. You are **paid** for it **by the market**, mainly through a premium price paid by the processor or retailer.

### Land tenure contract with environmental clauses

You enter into a land-tenure contract where you **commit to give particular attention to environmental aspects beyond legal requirements when producing on the leased land**. The landowner accepts a lower **lease payment** than for comparable land under usual land tenure agreements to compensate your additional efforts. In the contract, environmentally friendly management practices on the leased land are prescribed in order to maintain or improve environmental targets, e.g. water protection, landscape and biodiversity improvement or carbon sequestration or alternatively.

## Footnotes

1 This commissioned work relates to the CONSOLE project and information can be found at https://console-project.eu/.

2 The term ‘contract’ referred to throughout this paper is described by the World Bank (2020) as a legal agreement that states and explains a formal arrangement between different individuals or groups for providing a public asset or service, in which the private party bears significant risk and management responsibility. In this case, the public service is the management of AECPG such as air, water, landscapes etc.

3 Opportunistic behaviour where some land managers do not maximise their efforts for the benefit of the collective.

4 Full details are available at https://www.planetproof.eu/en/.

5 These are non-viable farms where neither a farmer nor their spouse has an off-farm job (Dillon et al. 2022).

6 Belgium, Finland, France, Italy and the Netherlands are included in this study.

7 Which is driven by high debt levels, strict regulations and low power in the market.

8 For example, learning new skills, self-challenge, professional pride, and moral/ethical reasons (Pavlis et al. 2016).

9 Based on the same data collected by D’Alberto et al. (2022) which are also used in this study.

10 The surveys were designed by D’Alberto et al. (2022).

11 In the case of the Netherlands, nearly 14,000 farmers were contacted and provided with details on how to complete the online survey.

12 This includes ‘agree’ and ‘strongly agree’ responses.

13 The chosen nationally representative datasets do not include forests.

14 Agreement includes respondents who either ‘agree’ or ‘strongly agree’ with a statement.

15 As previously discussed, Cullen et al. (2020). McGurk et al. (2020) and Cullen et al. (2021) find that farm size increases the likelihood of AES adoption.
